# Polymorphisms in the Insulin-Like Growth Factor Axis Are Associated with Gastrointestinal Cancer

**DOI:** 10.1371/journal.pone.0090916

**Published:** 2014-03-07

**Authors:** Jennie Ong, Jody Salomon, Rene H. M. te Morsche, Hennie M. J. Roelofs, Ben J. M. Witteman, Polat Dura, Martin Lacko, Wilbert H. M. Peters

**Affiliations:** 1 Department of Gastroenterology, Radboud University Nijmegen Medical Center, Nijmegen, the Netherlands; 2 Department of Gastroenterology, Hospital Gelderse Vallei, Ede, the Netherlands; 3 Department of Otorhinolaryngology, Head and Neck Surgery, Maastricht University Medical Center, Maastricht, the Netherlands; Albert Einstein College of Medicne, United States of America

## Abstract

**Introduction:**

Numerous factors influence the development of gastrointestinal (GI) cancer. The insulin-like growth factor (IGF) axis plays a role in embryonic and postnatal growth and tissue repair. Elevated levels of IGFs, low levels of IGF binding proteins (IGFBPs) and over-expression of IGF receptor (IGFR-I) were associated with several stages of cancer. Here, the prevalence of the single nucleotide polymorphisms (SNPs) rs6214 in the *IGF type I* (*IGF-I*) gene and rs6898743 in the growth hormone receptor (*GHR*) gene in patients with GI cancer and controls was studied.

**Materials & Methods:**

In this Dutch case-control study, DNA isolated from blood of 1,457 GI cancer patients; 438 patients with head and neck cancer (HNC), 475 with esophageal cancer (EC) and 544 with colorectal cancer (CRC) and 1,457 matched controls, was used to determine the rs6214 and rs6898743 genotypes by polymerase chain reaction. The association between these SNPs and GI cancer, HNC, esophageal adenocarcinoma (EAC), esophageal squamous-cell carcinoma (ESCC) and proximal or distal CRC was studied. Odds ratios (ORs) with 95% confidence interval (95% CI) were calculated via unconditional logistic regression.

**Results:**

Overall for GI cancer, the ORs for SNPs rs6214 and rs6898743 were approximately 1.0 (p-value>0.05), using the most common genotypes GG as reference. An OR of 1.54 (95% CI, 1.05–2.27) was found for EC for genotype AA of rs6214. The ORs for EAC were 1.45 (95% CI, 1.04–2.01) and 1.71 (95% CI, 1.10–2.68), for genotypes GA and AA, respectively. Genotype GC of rs6898743 showed an OR of 0.47 (95% CI, 0.26–0.86) for ESCC.

**Conclusion:**

The A allele of SNP rs6214 in the *IGF-I* gene was associated with EAC, and with HNC in women. The GC genotype of rs6898743 in the *GHR* gene was negatively associated with ESCC.

## Introduction

Worldwide, cancer is one of the most prevalent causes of death [1]. In 2011, the overall incidence of cancer in the Netherlands was 100,577 cases, approximately 20% did develop gastrointestinal (GI) cancer [2]. The number of new patients with GI cancer is increasing in the Netherlands over the past 20 years [2].

Ten to twenty percent of the GI cancers are hereditary [3]. There are several other factors that play a role in the development of GI cancer, including age, gender, smoking, physical activity and obesity [4], [5]. Also dietary factors, such as intake of alcohol, consumption of low amounts of vegetables and fruits, excessive consumption of red meat, and many other dietary factors may have an impact on the development of GI cancer [5]. In addition, risk modulating genetic factors with low penetrance, such as numerous single nucleotide polymorphisms (SNPs), may be involved in the development of GI cancer.

In this respect the insulin-like growth factor (IGF) axis may be of interest, since the evidence that it is involved in several stages of cancer is increasing [6]–[8]. The IGF axis plays a role in embryonic and postnatal growth, in tissue repair and many other important processes [6], [9], [10]. Elevated levels of IGFs, low levels of IGF binding proteins (IGFBPs) in the circulation and over-expression of IGF receptor type I (IGFR-I) were associated with cancer [7], [11]. *In vitro* studies indicated that several components of the IGF axis induce mitosis and prevents apoptosis in cancer cells [12], [13].

IGF is mainly produced by the liver and it is mediated by the growth hormone (GH) family. The predominant isoform of this somatotropin family is growth hormone 1 (GH1). The growth hormone releasing hormone (GHRH), secreted by the hypothalamus, regulates the production of GH in the adenohypophysis of the pituitary gland [14]. The GH binds to the growth hormone receptor (GHR) in the liver and other target tissues. This interaction promotes the synthesis of IGF-I and IGF-II [15].

IGF is able to bind to the members of the IGFBP family [16]. Most IGFs circulating in the blood stream are bound to IGFBPs with a high binding affinity, resulting in an increased half-life of the IGFs. The majority of IGFs in the circulation bind to IGFBP-3, an acid labile subunit (ALS) complex [17]. Numerous factors, including hormones, diet, ethnicity, age and sex, influence the levels of IGFs and IGFBP-3 in serum and tissues [18].

In addition to the IGFBP family members, IGF-I and IGF-II bind to the two types of IGF receptors, IGFR-I and IGFR-II. IGFR-I, a transmembrane tyrosine kinase receptor, is expressed throughout the human body. Both types of IGF are able to interact with this receptor with different binding affinities. Activation of the IGFR-I related pathway may lead to proliferation, differentiation, migration and prevention of a cell from going into apoptosis [8].

The function of IGFR-II, a cation-independent mannose-6-phosphate receptor, is less clear. This receptor interacts with IGF-I as well as with IGF-II. The binding of this receptor with IGF-I is weak, whereas IGFR-II has a high binding affinity for IGF-II. In contrast to IGFR-I, these interactions do not lead to activation of cell signaling pathways. The main function of this receptor is probably the sequestering of IGF-II and eventually degradation of this protein [9].

Several genetic variants in the components of the IGF axis were already investigated in association with cancer risk. McElholm et al. analyzed 102 SNPs in the IGF axis [17] and characterized three genetic variants that appeared to be associated with esophageal adenocarcinoma: rs6214 in the *IGF-I* gene, rs6898743 in the *GHR* gene and a CA-repeat in the promoter region (5′-UTR) of the *IGF-I* gene, the latter variant however being very rare. The association between the SNPs rs6214 and rs6898743 and other GI cancers was not investigated yet.

The SNP rs6214 (c.*2716G>A) resides on the *IGF-I* gene [19]. This gene is located on the long arm of chromosome 12 at position 23.2 and consists of six exons [20]. The SNP rs6214 is located in the three prime untranslated region (3′-UTR), which has an important role in the translation, localization and stability of mRNA [21]–[23]. In Europe, the allele frequencies of the G and A alleles are 55% and 45%, respectively [24].

The SNP rs6898743 (c.71-26648G>C) is an intronic SNP in the *GHR* gene, localized on chromosome 5 [17]. In the European population, approximately 75% and 25% are G and C alleles, respectively [25].

In this study, the prevalence of the SNPs rs6214 and rs6898743 in patients with GI cancer and controls were investigated in a Dutch population.

## Materials and Methods

### Study Population

For this population-based case-control study, whole blood or healthy tissue of Caucasian patients with GI cancer and controls was collected between 2002 and 2013 at several hospitals in the Netherlands. Enrollment criteria were that cases were included in the order of entry to the hospitals, and only patients who’s diagnosis was confirmed by a pathologist, were included in the study. All subsequent patients who were willing to participate, gave their written informed consent. The investigations were approved by the Medical Ethical Review Committees of the Maastricht University Medical Center and Radboud University Nijmegen Medical Center (RUNMC).

The blood and tissue samples of patients with CRC or EC were obtained from the RUNMC and the Gelderse Vallei Hospital, Ede. Patients with EC were also included at the Canisius-Wilhelmina Hospital Nijmegen and the Rijnstate Hospital, Arnhem. The blood samples of the healthy controls, for comparison with the CRC and EC patient groups, were recruited from the Nijmegen area, by advertisement in local papers. Blood of patients with HNC was collected at the University Hospital Maastricht. Healthy controls for this group of patients were recruited from the blood bank, Maastricht area. Controls were matched with the patients with respect to age and gender.

EC was classified by a pathologist as the histological subtypes esophageal squamous-cell carcinoma (ESCC) or esophageal adenocarcinoma (EAC). In patients with CRC, the location of the tumor was identified as being in the proximal (cecum, colon ascendens, colon transversum) or distal (colon descendens, sigmoid colon, rectum) part of the colon.

Blood was stored at −20°C at the laboratory of the Department of Gastroenterology, RUNMC. Controls were matched to patients by gender, age, ethnicity and recruiting area to create two comparable groups.

### DNA Isolation

In patients, DNA was isolated from blood or healthy tissue, obtained after resection of the tumor. In controls DNA was isolated from blood. For DNA isolation the High Pure PCR Template Preparation Kit (Roche Diagnostics GmbH, Mannheim, Germany) was used, according to the protocol of the manufacturer. DNA samples were stored at 4°C until use.

### Genotyping

The rs6214 and rs6898743 polymorphisms were established by means of real-time polymerase chain reaction (RT-PCR) techniques. A specific set of primers, which flank the region of the SNPs, was used to amplify the DNA. In the TaqMan assay, two labeled probes with a fluorophore at the 5′end and a quencher at the 3′ end, were added to the PCR mixture. Primers and probes were designed for each SNP, using the Beacon Designer 7.0 software (PREMIER Biosoft, Palo Alto, California, USA). Primers and TaqMan probes were prepared by Sigma-Aldrich Chemie BV. (Zwijndrecht, the Netherlands) and were checked for polymorphisms in their binding sites using SNPCheck version 3 (https://ngrl.manchester.ac.uk/SNPCheckV3/snpcheck.htm). The sequences of the primers and probes are given in Table 1. The 6-fluorescein amidite (6-FAM) and hexachloro-fluorescein amidite (HEX) were covalently bound to the probes for the most common alleles and the less common alleles, respectively. The Black Hole Quencher-1 (BHQ1), was used which was bound to the 3′-end of the TaqMan probe. The RT-PCRs were performed with the CFX96 Real-Time PCR detection system (Bio-Rad Laboratories Inc, Hercules, California, USA) and the results were analyzed with the data analysis software Bio-Rad CFX Manager 2.0 (Bio-Rad Laboratories Inc).

**Table 1:** Sequences of primers and probes, including the optimal annealing temperature and MgCl_2_-concentration.

SNP (*Gene*)	Primer/Probe	Sequence 5′ → 3′	Annealing Temperature	[MgCl_2_]
**rs6214 (** ***IGF-I*** **)**	F primer	CTCAACAAAACTTTATAGGCAGTC	60.0°C	5 mM
	R primer	GCAGTGCATCTTTCAGCTT		
	WT probe	CTGCAGACTTAAC**G**TGTTTTCTGTCATAG		
	VAR probe	CTGCAGACTTAAC**A**TGTTTTCTGTCATAG		
**rs6898743 (** ***GHR*** **)**	F primer	TACCATTTGCATGGAATATC	56.5°C	4 mM
	R primer	AGCTTAAATCTGTCCCTATC		
	WT probe	ATCACTTACCTTTTA**G**TGTATGTATGTACT		
	VAR probe	ATCACTTACCTTTTA**C**TGTATGTATGTACT		

F = forward; R = reverse; WT = wild type; VAR = variant; the bold underlined letters represent the wild type (most common) allele (Allele 1) and the variant allele (Allele 2) in the WT probe and VAR probe, respectively.

### Statistical Analyses

The results from the RT-PCRs, genotype and allele frequency distributions, were analyzed by using IBM SPSS Statistics software version 20 (International Business Machines Corp., Armonk, New York, USA).

The equality of the mean age was tested using the independent-samples t-test. In the analysis, age and gender were considered as confounders. Odds ratios (ORs) with their 95% confidence intervals (95% CIs) were calculated for the genotypes and for the alleles frequencies by performing unconditional as well as conditional logistic regression analyses. Homozygosity for the most common allele was taken as reference.

When the mean age between the patient (sub)group and the control group was significantly different, the ORs calculated with the unconditional logistic regression were corrected for this variable. Results with a *p*-value <0.05 were considered statistically significant.

## Results

The characteristics of the study population and the number of subjects per GI cancer subtype are summarized in Table 2. In this study, 1,457 patients with GI cancer and 1,457 controls were included, aged between 16 and 95 years. The mean age of the GI cancer patient and control groups was 63.8±11.5 and 62.4±11.3 years, respectively (*p*-value = 0.001).

**Table 2:** Characteristics of the study population.

Cancer subtype	Characteristics	Patients with GI cancer	Controls
**Head and neck**	n	438	438
	Male (%)	345 (78.8)	345 (78.8)
	Female (%)	93 (21.2)	93 (21.2)
	Mean age ± SD (years)^1^	60.9±11.3	56.5±6.7
**Esophageal**	n	475	475
	Male (%)	384 (80.8)	384 (80.8)
	Female (%)	81 (19.2)	81 (19.2)
	Mean age ± SD (years)	65.1±11.0	65.1±11.3
	Histological subtype		
	EAC	355	–
	ESCC	112	–
	Unknown	7	–
**Colorectal**	n	544	544
	Male (%)	325 (59.7)	325 (59.7)
	Female (%)	219 (40.3)	219 (40.3)
	Mean age ± SD (years)	65.0±11.7	64.7±12.5
	Location		
	Proximal	156	–
	Distal	366	–
	Unknown	21	–
**Total GI cancer**	n	1,457	1,457
	Male (%)	1,054 (72.3)	1,054 (72.3)
	Female (%)	403 (27.7)	403 (27.7)
	Mean age ± SD (years)^2^	63.8±11.5	62.4±11.3

n = number of patients or controls; GI, gastrointestinal; SD = standard deviation; EAC = esophageal adenocarcinoma; ESCC = esophageal squamous cell carcinoma;

1 *p*-value mean age = 0.000;

2 *p*-value mean age = 0.001.

In the sub-analyses, in which the association between the SNPs and the development of each cancer subtype was investigated, the mean age between the HNC patients and the corresponding control group was significantly different (p-value = 0.0001).

The genotype distribution for SNPs rs6214 and rs6898743 in patients with GI cancer and controls are presented in Table 3. For the SNPs rs6214 and rs6898743, the distribution of the genotypes in the overall GI cancer patient group, in the GI cancer sub-groups as well as in the control groups did not deviate from the Hardy-Weinberg equilibrium (all p-values >0.05), except for the ESCC patient sub-group, which showed a *p*-value of 0.003 for SNP rs6898743.

**Table 3:** Genotype distribution in patients with gastrointestinal cancer and controls.

SNP (*Gene*)	Genotype	Patients with GI cancer	Controls	OR (95% CI) **	*p*-value
		n (%)*	n (%)*		
**rs6214 (** ***IGF-I*** **)**	**GG**	491 (34.0)	528 (36.3)	Reference	
	**GA**	709 (49.1)	706 (48.5)	1.08 (0.92–1.27)	0.339
	**AA**	245 (17.0)	221 (15.3)	1.20 (0.96–1.49)	0.111
**rs6898743 (** ***GHR*** **)**	**GG**	844 (58.5)	859 (59.0)	Reference	
	**GC**	518 (35.9)	522 (35.9)	1.01 (0.87–1.18)	0.896
	**CC**	81 (5.6)	75 (5.2)	1.10 (0.79–1.53)	0.558

*Note that some patients or controls are missing because of failure of the genotyping.

**Adjusted for age.

GI, gastrointestinal; OR, odds ratio; CI, confidence interval.

For both SNPs, no associations with GI cancer were seen (ORs between 1.01 and 1.20, *p*-values >0.05), except for a significant over-expression of the GA genotype of rs6214 in female patients (OR 1.38, 95% CI 1.01–1.87).

Table 4 summarizes the genotype distribution and the ORs (95% CI) for SNPs rs6214 and rs6898743 per cancer subtype. In this sub-analysis, a significant association with EC (OR 1.54, 95% CI, 1.05–2.27) was found for genotype AA of rs6214 (*p*-value = 0.029), whereas the GA genotype showed a non-significant OR of 1.27 (95% CI 0.96–1.69). The A-allele was significantly more present in the patient group (p-value = 0.021). Analyzed according to gender, genotype AA of rs6214 in men showed an even higher OR of 1.68 (95% CI, 1.09–2.59) with a p-value of 0.019.

**Table 4:** Genotype distribution and ORs (95% CI) per cancer subtype, for the genotypes of SNPs rs6214 and rs6898743.

Cancer subtype	SNP (*Gene*)	Genotype	Patients	Controls	OR (95% CI)	*p*-value
			n (%)	n (%)		
**HNC**	**rs6214 (** ***IGF-I*** **)**	**GG**	153 (35.3)	147 (33.6)	Reference	
		**GA**	210 (48.5)	214 (49.0)	0.91 (0.67–1.24)*	0.550
		**AA**	70 (16.2)	76 (17.4)	0.88 (0.58–1.32)*	0.527
	**rs6898743 (** ***GHR*** **)**	**GG**	246 (56.9)	263 (60.0)	Reference	
		**GC**	164 (38.0)	156 (35.6)	1.08 (0.81–1.44)*	0.588
		**CC**	22 (5.1)	19 (4.3)	1.16 (0.61–2.23)*	0.654
**EC**	**rs6214 (** ***IGF-I*** **)**	**GG**	155 (33.0)	187 (39.5)	Reference	
		**GA**	230 (49.0)	221 (46.6)	1.27 (0.96–1.69)	0.096
		**AA**	84 (17.9)	66 (13.9)	**1.54 (1.05–2.27)**	**0.029**
	**rs6898743 (** ***GHR*** **)**	**GG**	271 (57.9)	273 (57.6)	Reference	
		**GC**	164 (35.0)	181 (38.2)	0.92 (0.70–1.20)	0.524
		**CC**	33 (7.1)	20 (4.2)	1.67 (0.93–2.99)	0.085
**CRC**	**rs6214 (** ***IGF-I*** **)**	**GG**	183 (33.7)	194 (35.7)	Reference	
		**GA**	269 (49.5)	271 (49.8)	1.04 (0.80–1.36)	0.750
		**AA**	91 (16.8)	79 (14.5)	1.21 (0.84–1.74)	0.311
	**rs6898743 (** ***GHR*** **)**	**GG**	327 (60.2)	323 (59.4)	Reference	
		**GC**	190 (35.0)	185 (34.0)	1.02 (0.79–1.32)	0.868
		**CC**	26 (4.8)	36 (6.6)	0.73 (0.43–1.23)	0.235

*Adjusted for age; OR, odds ratio; CI, confidence interval; HNC, head and neck cancer; EC, esophageal carcinoma; CRC, colorectal cancer.

No significant associations were found between patients with HNC or CRC and controls for the genotypes of both SNPs. However, for HNC in women an OR of 2.19 (95% CI, 1.09–4.39) was found for the GA genotype of rs6214, whereas an OR of 0.08, 95% CI, 0.01–0.65 was found for the CC genotype of SNP rs6898743. The ORs calculated for the rs6214 and rs6898743 genotypes, per histological subtype of EC are shown in Table 5. An OR of 1.45 (95% CI, 1.04–2.01) was found for GA heterozygotes of rs6214 in EAC (p-value = 0.028), whereas homozygotes for the variant allele AA showed an OR of 1.71 (95% CI, 1.10–2.68; p-value = 0.018). In men with genotype AA of rs6214, the association was even stronger (OR 1.87, 95% CI, 1.14–3.07; *p = *0.013). The A-allele was significantly more present in the EAC patient group as well as in the male sub-group (p-values 0.008 and 0.009, respectively).

**Table 5:** Genotype distribution and ORs (95% CI) for the genotypes of SNPs rs6214 and rs6898743 in patients with EAC, ESCC, proximal CRC or distal CRC.

Cancer subtype	SNP (*Gene*)	Genotype	Patients	Controls	OR (95% CI)	*p*-value
			n (%)	n (%)		
**EAC**	**rs6214 (** ***IGF-I*** **)**	**GG**	115 (32.9)	147 (42.1)	Reference	
		**GA**	171 (48.9)	154 (44.1)	**1.45 (1.04–2.01)**	**0.028**
		**AA**	64 (18.3)	48 (13.8)	**1.71 (1.10–2.68)**	**0.018**
	**rs6898743 (** ***GHR*** **)**	**GG**	189 (54.2)	199 (57.0)	Reference	
		**GC**	137 (39.3)	133 (38.1)	1.10 (0.81–1.51)	0.540
		**CC**	23 (6.6)	17 (4.9)	1.43 (0.74–2.78)	0.289
**ESCC**	**rs6214 (** ***IGF-I*** **)**	**GG**	39 (34.8)	35 (31.3)	Reference	
		**GA**	54 (48.2)	60 (53.6)	0.80 (0.44–1.45)	0.450
		**AA**	19 (17.0)	17 (15.2)	1.20 (0.53–2.74)	0.661
	**rs6898743 (** ***GHR*** **)**	**GG**	78 (69.6)	66 (58.9)	Reference	
		**GC**	25 (22.3)	43 (38.4)	**0.47 (0.26–0.86)**	**0.014**
		**CC**	9 (8.0)	3 (2.7)	2.54 (0.66–9.81)	0.175
**Proximal CRC**	**rs6214 (** ***IGF-I*** **)**	**GG**	51 (32.7)	55 (35.3)	Reference	
		**GA**	82 (52.6)	74 (47.4)	1.20 (0.73–1.96)	0.481
		**AA**	23 (14.7)	27 (17.3)	0.94 (0.48–1.86)	0.868
	**rs6898743 (** ***GHR*** **)**	**GG**	99 (63.5)	90 (57.7)	Reference	
		**GC**	54 (34.6)	59 (37.8)	0.84 (0.53–1.34)	0.470
		**CC**	3 (1.9)	7 (4.5)	0.39 (0.10–1.58)	0.188
**Distal CRC**	**rs6214 (** ***IGF-I*** **)**	**GG**	123 (33.6)	131 (35.8)	Reference	
		**GA**	180 (49.2)	186 (50.8)	1.03 (0.74–1.41)	0.879
		**AA**	63 (17.2)	49 (13.4)	1.37 (0.87–2.15)	0.173
	**rs6898743 (** ***GHR*** **)**	**GG**	217 (59.3)	220 (60.1)	Reference	
		**GC**	126 (34.4)	119 (32.5)	1.09 (0.80–1.50)	0.581
		**CC**	23 (6.3)	27 (7.4)	0.90 (0.50–1.62)	0.721

EAC, esophageal adenocarcinoma; ESCC, esophageal squamous cell carcinoma; CRC, colorectal cancer.

For SNP rs6898743, an inverse association with ESCC was noticed for genotypes GC (OR 0.47, 95% CI, 0.26–0.86) with a *p*-value of 0.014. In contrast, the homozygote variant CC genotype revealed an OR of 2.54 (95% CI, 0.66–9.81). However in these CC subgroups the number of individuals are very small. Likewise, a significant inverse association with ESCC was found in men with genotype GC (OR 0.31, 95% CI, 0.15–0.66; *p* = 0.002). No significant associations were found when CRC patients were analyzed according to tumor localization, either proximal or distal (Table 5).

**Figure 1:**
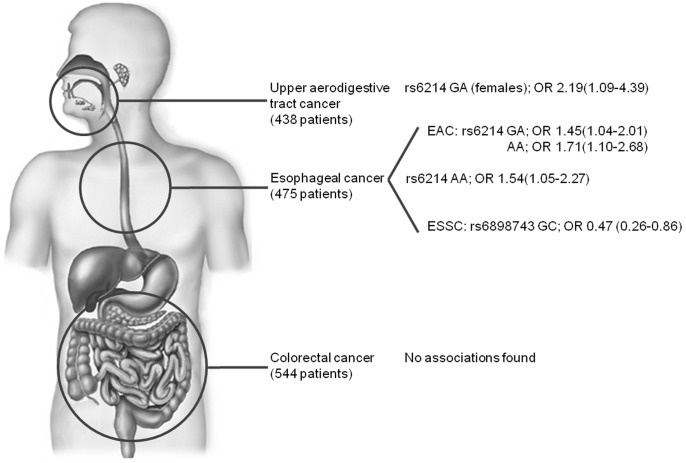
Associations of the SNPs rs6214 and rs6898743 with gastrointestinal cancer. Only significant associations are visualized. Associations are given as follows: rs number of the SNP, genotype, odds ratio with corresponding 95% confidence interval. The homozygous most common genotype is taken as reference. EAC, esophageal adenocarcinoma; ESCC, esophageal squamous cell carcinoma.

The main results of this study are visualized in Figure 1.

## Discussion

This population-based case-control study was performed to investigate the role of the IGF associated SNPs rs6214 and rs6898743 in the development of gastrointestinal cancer. The SNPs rs6214 and rs6898743 were not found associated with GI cancer, however in GI cancer subgroups, some interesting associations were noticed.

Genotype AA of rs6214 in the *IGF-I* gene was associated with EC, but this risk modifying effect of the rs6214 A allele was mainly the result of an association with the histological subgroup of EAC; individuals bearing the GA or AA genotypes showed relative risks of 1.45 and 1.71, respectively. The SNP rs6214 has been investigated in several previous studies on GI cancer risk. Feik et al. reported a significantly higher risk of CRC for carriers of genotype AA [26]. A similar trend was seen in our study but no significance was reached. This difference could be explained by the fact that the study population of Feik et al. only contained 178 CRC patients. Moreover, their ORs were adjusted for gender, age and body mass index (BMI), whereas BMI was not involved in our study.

McElholm et al. reported a significant decreased risk of Barrett’s esophagus (BE) for the AA genotype of rs6214 in an Irish study population (OR = 0.43, 95% CI 0.24–0.75) [17]. BE is an abnormal replacement in the esophagus of squamous epithelium by specialized columnar-lined epithelium, and is one of the main risk factors for EAC [27]. Therefore it is surprising that we found a significant inverse association with EAC for the rs6214 GA and AA genotypes.

Two main explanations may be given: first, the patient group with BE and the control group in the study of McElholm et al. were relatively small (n = 207 and n = 244, respectively). More important however, is the large difference in allele frequencies, being 48% and 52% for the rs6214 G and A alleles as reported by McElholm et al. in their controls, whereas values of 57% and 43% were reported for Europeans in the HAPMAP database [24]. The latter values are in good agreement with our control allele frequency data (60.5% G and 39.5% A allele).

The role of the genetic variant of rs6214 in pancreatic cancer in a Japanese population was assessed by Nakao et al. [28]. Using AA as the reference genotype, no association was found between rs6214 and pancreatic cancer in the overall analysis, whereas 20 years old carriers of the GA and GG genotypes with a BMI ≥25 kg/m^2^ showed an increased risk for pancreatic cancer. However, the number of patients included in this sub-category was very low and thus reliability is limited.

High plasma levels of IGF-I were suggested to be a risk for various cancers, including CRC [29], [30]. Therefore, Canzian et al., Al-Zahrani et al. and D’Aloisio et al. investigated the role of the rs6214 SNP on the circulating IGF-I levels in Caucasians [20], [31], [32]. However, inconsistent results were obtained. In the study of D’Aloisio et al., the AA genotype appeared to be associated with high mean IGF-I plasma levels, whereas the other two studies did not find an association between this SNP and circulating IGF-I levels.

The SNP rs6898743 has not been extensively investigated so far. Carriers of the CC genotypes were found inversely associated with EAC by McElholm et al. [17], whereas our results showed a tendency for the opposite. It was also remarkable that the allele frequencies in the Irish study population as reported by McElholm et al. [17] (19% G and 81% C for controls) were completely different from the HAPMAP frequencies in the European population [25] (77.9% G and 22.1% C). Our data of 76.9% G allele and 23.1% C allele in controls completely fit with the reported HAPMAP data for Europeans [25].

Some interesting gender related differences were found; in women, genotype GA of SNP rs6214 was significantly associated with GI- and HNC, whereas homozygosity for the variant C allele of rs6898743 was inversely associated with HNC. In men, exactly opposite effects were noticed, however results here are not significant. It should be noticed however that the number of female patients in the sub-group of HNC patients was small (n = 93), which made the significance disputable.

The strength of the present study is that the patients and controls were successfully matched by sex, race, recruiting area and also largely by age. This enabled an assessment of association between the SNPs and the outcomes, without being influenced by these confounders. Moreover, the overall study population was large.

This study also has its limitations. First of all, the matching by age was not succeeded completely, as the mean ages between the GI- and HNC patient and control groups differed significantly. Therefore, unconditional logistic regression was used which theoretically creates a lower power compared to conditional logistic regression. Secondly, the ORs calculated in the sub-analyses were not adjusted for multiple testing. This might be advisable since multiple testing may increase the change to find a significant association and thus creates bias. Thirdly, in this study no information on smoking habits, alcohol consumption, diet and other lifestyle factors is available. Furthermore, obesity of which BMI and hip-to-waist ratio are suitable indicators, is an important confounder for GI cancer [33]. Obesity may influence the risk of colorectal cancer, and excess of body fat and fat distribution also appeared to be related to EAC [33]. Co-morbidity, especially early diagnosed diabetes mellitus, is associated with various cancers, including cancers of the colorectum [34]. Moreover, in analyzing the relationship between polymorphisms in the components of the IGF axis and cancer in women, the use of birth control pills needs to be taken into consideration, since the sex hormone estradiol has a direct, as well as an indirect influence on the production of IGF-I in estrogen-responsive tissues and the liver [35]. Other limitation of this study is that the study populations in the sub-analyses were relatively small, and that only one control was matched with one patient.

In conclusion, the A allele of SNP rs6214 of the *IGF-I* gene is associated with esophageal cancer. This association might be mainly the result of effects on the histological subtype esophageal adenocarcinoma in men. Furthermore, women with the A allele of SNP rs6214 may have an increased risk for developing GI cancer, more in particular HNC. These SNPs could be used as markers to assess the cancer risk for these (histological) subtypes.

## References

[1] IARC, Lyon, France. GLOBOCAN 2008. Section of Cancer Information. Available: http://globocan.iarc.fr/factsheets/populations/factsheet.asp?uno=900.

[2] Integraal Kankercentrum Nederland. Cijfers over Kanker. Available: http://www.cijfersoverkanker.nl/selecties/dataset_1/img51729ed128e20.

[3] Ho SM, Ho JW, Chan CL, Kwan K, Tsui YK (2003) Decisional consideration of hereditary colon cancer genetic test results among Hong Kong chinese adults. Cancer Epidemiol Biomarkers Prev 12: 426–432. 12750237

[4] Zhivotovskiy AS, Kutikhin AG, Azanov AZ, Yuzhalin AE, Magarill YA, et al (2012) Colorectal cancer risk factors among the population of South-East Siberia: a case-control study. Asian Pac J Cancer Prev 13: 5183–5188. 23244132 10.7314/apjcp.2012.13.10.5183

[5] Haggar FA, Boushey RP (2009) Colorectal cancer epidemiology: incidence, mortality, survival, and risk factors. Clin Colon Rectal Surg 22: 191–197. 21037809 10.1055/s-0029-1242458 PMC2796096

[6] Chhabra Y, Waters MJ, Brooks AJ (2010) Role of the growth hormone–IGF-1 axis in cancer. Expert Rev Endocrinol Metab 6: 71–84. 10.1586/eem.10.73 30764037

[7] Samani AA, Yakar S, LeRoith D, Brodt P (2007) The role of the IGF system in cancer growth and metastasis: overview and recent insights. Endocr Rev 28: 20–47. 16931767 10.1210/er.2006-0001

[8] LeRoith D, Roberts CT Jr (2003) The insulin-like growth factor system and cancer. Cancer Lett 195: 127–137. 12767520 10.1016/s0304-3835(03)00159-9

[9] Denley A, Cosgrove LJ, Booker GW, Wallace JC, Forbes BE (2005) Molecular interactions of the IGF system. Cytokine Growth Factor Rev 16: 421–439. 15936977 10.1016/j.cytogfr.2005.04.004

[10] Estivariz CF, Ziegler TR (1997) Nutrition and the insulin-like growth factor system. Endocrine 7: 65–71. 9449035 10.1007/BF02778066

[11] Yu H, Rohan T (2000) Role of the insulin-like growth factor family in cancer development and progression. J Natl Cancer Inst 92: 1472–1489. 10995803 10.1093/jnci/92.18.1472

[12] Gallagher EJ, LeRoith D (2011) Minireview: IGF, Insulin, and Cancer. Endocrinology 152: 2546–2551. 21540285 10.1210/en.2011-0231

[13] Macaulay VM (1992) Insulin-like growth factors and cancer. Br J Cancer 65: 311–320. 1313689 10.1038/bjc.1992.65 PMC1977607

[14] Ionescu M, Frohman LA (2006) Pulsatile secretion of growth hormone (GH) persists during continuous stimulation by CJC-1295, a long-acting GH-releasing hormone analog. J Clin Endocrinol Metab 91: 4792–4797. 17018654 10.1210/jc.2006-1702

[15] Kaskel F (2003) Chronic renal disease: a growing problem. Kidney Int 64: 1141–1151. 12911573 10.1046/j.1523-1755.2003.00194.x

[16] Hwa V, Oh Y, Rosenfeld RG (1999) The insulin-like growth factor-binding protein (IGFBP) superfamily. Endocr Rev 20: 761–787. 10605625 10.1210/edrv.20.6.0382

[17] McElholm AR, McKnight AJ, Patterson CC, Johnston BT, Hardie LJ, et al (2010) A population-based study of IGF axis polymorphisms and the esophageal inflammation, metaplasia, adenocarcinoma sequence. Gastroenterology 139: 204–212. 20403354 10.1053/j.gastro.2010.04.014

[18] Hernandez W, Grenade C, Santos ER, Bonilla C, Ahaghotu C, et al (2007) IGF-1 and IGFBP-3 gene variants influence on serum levels and prostate cancer risk in African-Americans. Carcinogenesis 28: 2154–2159. 17724372 10.1093/carcin/bgm190

[19] Canzian F, McKay JD, Cleveland RJ, Dossus L, Biessy C, et al (2006) Polymorphisms of genes coding for insulin-like growth factor 1 and its major binding proteins, circulating levels of IGF-I and IGFBP-3 and breast cancer risk: results from the EPIC study. Br J Cancer 94: 299–307. 16404426 10.1038/sj.bjc.6602936 PMC2361124

[20] Bonapace G, Concolino D, Formicola S, Strisciuglio P (2003) A novel mutation in a patient with insulin-like growth factor 1 (IGF1) deficiency. J Med Genet 40: 913–917. 14684690 10.1136/jmg.40.12.913 PMC1735341

[21] Zhao J, Xiong DH, Guo Y, Yang TL, Recker RR, et al (2007) Polymorphism in the insulin-like growth factor 1 gene is associated with age at menarche in caucasian females. Hum Reprod 22: 1789–1794. 17376820 10.1093/humrep/dem052

[22] Kim HJ, Kim SK, Park HJ, Chung JH, Chun J, et al (2012) Polymorphisms of IGFI contribute to the development of ischemic stroke. Exp Ther Med 3: 93–98. 22969851 10.3892/etm.2011.372 PMC3438751

[23] Chatterjee S, Pal JK (2009) Role of 5′- and 3′-untranslated regions of mRNAs in human diseases. Biol Cell 101: 251–262. 19275763 10.1042/BC20080104

[24] NCBI. Reference SNP (refSNP) Cluster Report: rs6214. Available: http://www.ncbi.nlm.nih.gov/projects/SNP/snp_ref.cgi?rs=6214.

[25] NCBI. Reference SNP (refSNP) Cluster Report: rs6898743. Available: http://www.ncbi.nlm.nih.gov/projects/SNP/snp_ref.cgi?rs=6898743.

[26] Feik E, Baierl A, Hieger B, Führlinger G, Pentz A, et al (2010) Association of IGF1 and IGFBP3 polymorphisms with colorectal polyps and colorectal cancer risk. Cancer Causes Control 21: 91–97. 19784788 10.1007/s10552-009-9438-4

[27] Estores D, Velanovich V (2013) Barrett esophagus: epidemiology, pathogenesis, diagnosis, and management. Curr Probl Surg 50: 192–226. 23601575 10.1067/j.cpsurg.2013.01.004

[28] Nakao M, Hosono S, Ito H, Watanabe M, Mizuno N, et al (2011) Interaction between IGF-1 polymorphisms and overweight for the risk of pancreatic cancer in Japanese. Int J Mol Epidemiol Genet 2: 354–366. 22199998 PMC3243451

[29] Ma J, Pollak M, Giovannucci E, Chan JM, Tao Y, et al.. (2000) A prospective study of plasma levels of insulin-like growth factor I (IGF-I) and IGF-binding protein-3, and colorectal cancer risk among men. Growth Horm IGF Res 10 Suppl A: S28–29. 10.1016/s1096-6374(00)90013-3 10984282

[30] Giovannucci E, Pollak MN, Platz EA, Willett WC, Stampfer MJ, et al (2000) A prospective study of plasma insulin-like growth factor-1 and binding protein-3 and risk of colorectal neoplasia in women. Cancer Epidemiol Biomarkers Prev 9: 345–349. 10794477

[31] Al-Zahrani A, Sandhu MS, Luben RN, Thompson D, Baynes C, et al (2006) IGF1 and IGFBP3 tagging polymorphisms are associated with circulating levels of IGF1, IGFBP3 and risk of breast cancer. Hum Mol Genet 15: 1–10. 16306136 10.1093/hmg/ddi398

[32] D’Aloisio AA, Schroeder JC, North KE, Poole C, West SL, et al (2009) IGF-I and IGFBP-3 polymorphisms in relation to circulating levels among African American and Caucasian women. Cancer Epidemiol Biomarkers Prev 18: 954–966. 19240240 10.1158/1055-9965.EPI-08-0856 PMC2896274

[33] Boeing H (2013) Obesity and cancer - The update 2013. Best Pract Res Clin Endocrinol Metab 27: 219–227. 23731883 10.1016/j.beem.2013.04.005

[34] Renehan AG, Frystyk J, Flyvbjerg A (2006) Obesity and cancer risk: the role of the insulin-IGF axis. Trends Endocrinol Metab 17: 328–336. 16956771 10.1016/j.tem.2006.08.006

[35] Kelemen LE, Sellers TA, Vachon CM (2008) Can genes for mammographic density inform cancer aetiology? Nat Rev Cancer 8: 812–823. 18772892 10.1038/nrc2466 PMC2818036

